# TADB 3.0: an updated database of bacterial toxin–antitoxin loci and associated mobile genetic elements

**DOI:** 10.1093/nar/gkad962

**Published:** 2023-10-28

**Authors:** Jiahao Guan, Yongkui Chen, Ying-Xian Goh, Meng Wang, Cui Tai, Zixin Deng, Jiangning Song, Hong-Yu Ou

**Affiliations:** State Key Laboratory of Microbial Metabolism, Joint International Laboratory on Metabolic & Developmental Sciences, School of Life Sciences & Biotechnology, Shanghai Jiao Tong University, Shanghai 200240, China; State Key Laboratory of Microbial Metabolism, Joint International Laboratory on Metabolic & Developmental Sciences, School of Life Sciences & Biotechnology, Shanghai Jiao Tong University, Shanghai 200240, China; State Key Laboratory of Microbial Metabolism, Joint International Laboratory on Metabolic & Developmental Sciences, School of Life Sciences & Biotechnology, Shanghai Jiao Tong University, Shanghai 200240, China; State Key Laboratory of Microbial Metabolism, Joint International Laboratory on Metabolic & Developmental Sciences, School of Life Sciences & Biotechnology, Shanghai Jiao Tong University, Shanghai 200240, China; State Key Laboratory of Microbial Metabolism, Joint International Laboratory on Metabolic & Developmental Sciences, School of Life Sciences & Biotechnology, Shanghai Jiao Tong University, Shanghai 200240, China; State Key Laboratory of Microbial Metabolism, Joint International Laboratory on Metabolic & Developmental Sciences, School of Life Sciences & Biotechnology, Shanghai Jiao Tong University, Shanghai 200240, China; Biomedicine Discovery Institute and Department of Biochemistry and Molecular Biology, Monash University, Melbourne, VIC 3800, Australia; Monash Data Futures Institute, Monash University, Melbourne, VIC 3800, Australia; State Key Laboratory of Microbial Metabolism, Joint International Laboratory on Metabolic & Developmental Sciences, School of Life Sciences & Biotechnology, Shanghai Jiao Tong University, Shanghai 200240, China

## Abstract

TADB 3.0 (https://bioinfo-mml.sjtu.edu.cn/TADB3/) is an updated database that provides comprehensive information on bacterial types I to VIII toxin–antitoxin (TA) loci. Compared with the previous version, three major improvements are introduced: First, with the aid of text mining and manual curation, it records the details of 536 TA loci with experimental support, including 102, 403, 8, 14, 1, 1, 3 and 4 TA loci of types I to VIII, respectively; Second, by leveraging the upgraded TA prediction tool TAfinder 2.0 with a stringent strategy, TADB 3.0 collects 211 697 putative types I to VIII TA loci predicted in 34 789 completely sequenced prokaryotic genomes, providing researchers with a large-scale dataset for further follow-up analysis and characterization; Third, based on their genomic locations, relationships of 69 019 TA loci and 60 898 mobile genetic elements (MGEs) are visualized by interactive networks accessible through the user-friendly web page. With the recent updates, TADB 3.0 may provide improved *in silico* support for comprehending the biological roles of TA pairs in prokaryotes and their functional associations with MGEs.

## Graphical Abstract

**Graphical Abstract:**
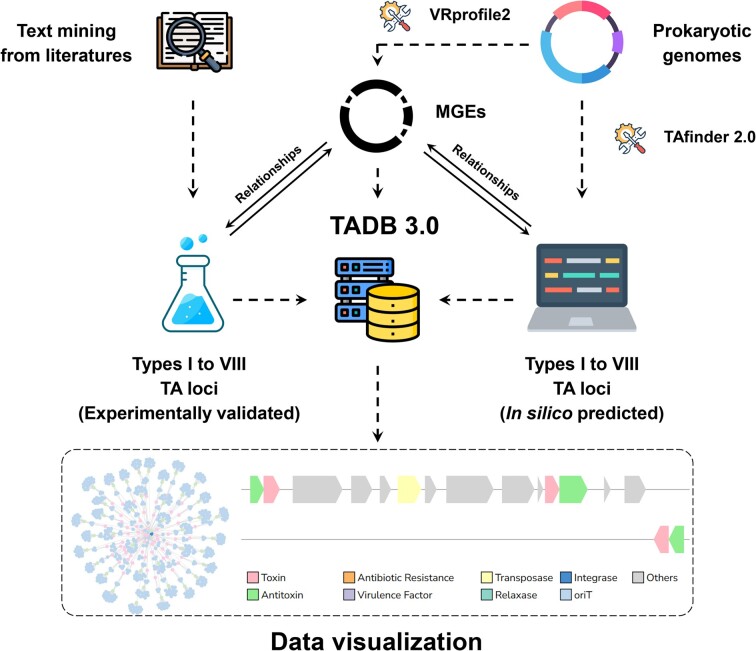

## Introduction

Toxin-antitoxin (TA) loci are small genetic modules commonly found in prokaryote genomes, whose prevalence and diversity have been extensively documented in numerous chromosomes and plasmids (1). Typically, a TA locus consists of a pair of genes: one encodes a stable toxin that inhibits essential cellular functions, and another encodes a labile antitoxin that neutralizes the activity of its cognate toxin. TA systems have been found to participate in various biological processes essential for bacterial survival and adaptation, such as stress response (2), bacteriophage defense (3), bacterial virulence (4), antimicrobial resistance (5) and plasmid stability (6). The known TA loci can be classified into eight types based on the nature and mode of action of the antitoxins, namely types I to VIII (7,8). The majority of described toxins are proteins (types I to VII), while the toxins are RNAs for type VIII TA pairs. Antitoxins are proteins for types II and IV to VII TA pairs but are RNAs in types I, III and VIII TA pairs.

Bacterial mobile genetic elements (MGEs), such as prophages, genomic islands (GIs), integrative and conjugative elements (ICEs), and plasmids, often harbor genes conferring selective advantages, including antibiotic resistance determinants and virulence factors, thereby shaping bacterial evolution and dissemination of these traits (9,10). Notably, growing evidence indicates the significant associations between TA loci and MGEs, highlighting their functional interplay (11–13). For example, the type II TA pairs have been well-documented to be an addiction module to ensure the maintenance of plasmids during bacterial replication (6,14). Recently, a plasmid-carrying *prpT/prpA* locus of *Pseudoalteromonas rubra* was found to control the plasmid copy number (15). TA loci have also been identified in relation to the genetic stability of other MGEs such as genomic islands (13), prophages (16), integrative and conjugative elements (17), and integrons (18). Interestingly, a *creTA* type VIII TA locus was proposed to maintain CRISPR immunity by making cells addicted to CRISPR-Cas (19). Therefore, understanding the relationships between TA loci and MGEs might shed light on the mechanisms underlying horizontal gene transfer, bacterial survival under environmental stress and bacterial adaptation to changing environments.

The field of TA systems has witnessed remarkable progress over the years, with various databases providing valuable resources for characterizing these systems. TASmania (20) is a database archiving *in silico* discovered types I to IV TA loci using curated HMM models. The T1TAdb database (21) specifically collected information on type I TA loci, including annotations of toxin peptides and antitoxin RNA molecules. The RASTA online tool (22) searches the conserved functional domains of individual toxins or antitoxin proteins, but its website maintenance was discontinued in 2011. In the year of 2011, we proposed the open-access database TADB1.1 (23) archiving both the experimentally validated and *in silico* predicted type II TA pairs. In 2018, we released the updated database TADB 2.0 (24), which systematically archives both the updated experimentally validated and *in silico* predicted type II TA loci, along with an online tool TAfinder for the identification of type II TA loci in bacterial genomes. Since then, a rapidly increasing number of new type II and other types of TA pairs have been characterized experimentally. Furthermore, although the interplay between TA loci and MGEs plays a crucial role in bacterial survival and evolution, existing TA-related databases often lack comprehensive information on their associations. Thus, the demand for a major database update became urgent.

Here, we report the release of TADB version 3.0, which provides an up-to-date comprehensive deposition of experimentally supported types I to VIII TA pairs. Meanwhile, we have upgraded TAfinder to version 2.0, considerably expanding its capabilities to predict a wide range of types I to VIII TA loci in completely sequenced bacterial and archaeal genomes. Furthermore, TADB 3.0 provides a user-friendly webpage to interrogate and display the relationships of TA loci and MGEs based on their genomic locations. We expect that TADB 3.0 will provide better support for exploring the diversity of TA pairs and offering crucial insights into the intricate relationships between TA loci and MGEs.

## Materials and methods

### Data updated by text mining and manual curation

Following a meticulous manual curation process of literatures from PubMed using the keyword ‘toxin–antitoxin system’, TADB 3.0 has incorporated 335 TA system-related publications published since 2017, bringing the total papers in the database to 921. After text mining and manual curations of these papers, the number of experimentally validated type II TA loci has significantly expanded from 105 to 403 in TADB 3.0. Moreover, this updated database includes 102, 8, 14, 1, 1, 3 and 4 experimentally validated TA loci of types I, III, IV, V, VI, VII and VIII, respectively (Supplementary Table S1). Notably, the families and genomic locations of 36 type I TA loci were collected with reference to T1TAdb (21), and hyperlinks to T1TAdb entries were built for these type I TA loci. In addition, using a rigorous prediction strategy, TADB 3.0 also records 16 009, 168 794, 55, 16 685, 2 786, 2, 388 and 6 978 *in silico* predicted TA loci of types I to VIII, respectively, as elaborated in the following section. Consequently, the TADB 3.0 database now archives a total of 16 111, 169 197, 63, 16 699, 2 787, 3, 391 and 6 982 TA loci, all accessible within the database (Supplementary Table S1).

**Figure 1.**
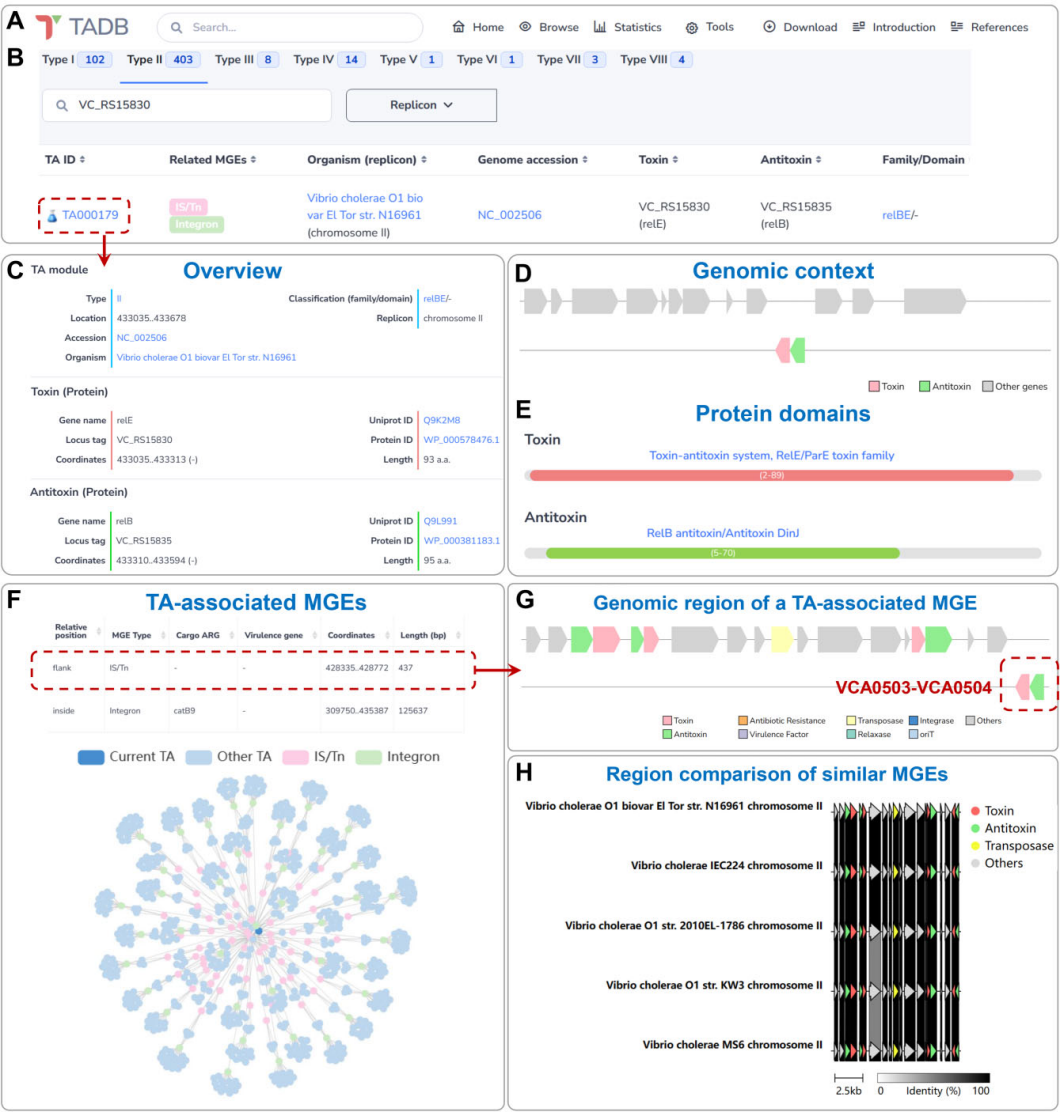
Updated web interface of TADB 3.0. (**A**) The functional modules of TADB 3.0 on the home page. (**B**) The ‘Browse’ web page displaying information on the *relBE* family TA locus (*VCA0503/VC_RS15830-VCA0504/VC_RS15835*) in *Vibrio cholerae* O1 biovar El Tor str. N16961. (**C**) An overview of the features of TA locus *VCA0503-VCA0504* on the detailed information page. (**D**) Visualization of the genomic context of TA locus by SVGene. (**E**) Protein domains of the toxin *VCA0503* and the antitoxin *VCA0504* predicted by InterProScan. (**F**) Interactive networks highlighting the MGEs associated with the *VCA0503-VCA0504* TA locus identified by VRprofile2. Similar MGEs were defined as MGEs with pairwise Mash distances lower than 0.01. (**G**) Visualization of the gene structures around the TA-associated MGEs by SVGene using an IS element flanked by TA locus *VCA0503-VCA0504* as an example. (**H**) Partial view of the comparison of similar MGEs by clinker and clustermap.js.

Similar to the previous version, TADB 3.0 continues to utilize the PostgreSQL relational database, PHP data pipeline and HTML web interfaces for organizing the TA loci data. In addition, the Bootstrap library (https://getbootstrap.com/) and data visualization library powered by JavaScript, such as ECharts (25) and SVGene (https://github.com/kblin/svgene), are added to various web interfaces for more user-friendly browsing. Illustrations of the TADB 3.0 data presentation interfaces are provided in Figure 1 and Supplementary Figure S1. The domains of toxin and antitoxin proteins were predicted by InterProScan (26). For each toxin or antitoxin, similar proteins were identified using NCBI BLASTp (27). Multiple sequence alignments of these similar proteins were performed by Clustal Omega (28) and subsequently visualized by the R package msa (29). For protein toxins and antitoxins, the experimentally determined and predicted three-dimensional structures were obtained from the RCSB Protein Data Bank (PDB) (https://www.rcsb.org/) (30) and AlphaFold Protein Structure Database (31), respectively. These structures were then visualized using PDBe Mol* (32). In the case of RNA toxins and antitoxins, the RNA secondary structures were predicted by RNAfold (33) and visualized by VARNA (34).

### Prediction of types I to VIII TA loci in completely sequenced prokaryotic genomes using TAfinder 2.0

The TAfinder tool, initially introduced in the TADB 2.0 database (24), is designed to predict type II TA loci through the search of homologs for toxin or antitoxin proteins by utilizing NCBI BLASTp (27) and HMMER3 (35). Types II to VII TA loci typically consist of two tandem genes located on the same DNA strand, coding for cognate protein partners. In contrast, the type I TA system consists of a toxin gene and an antitoxin RNA that are located on the opposite DNA strands. The type VIII TA system consists of a toxin RNA and an antitoxin RNA, which are located either on the same strand or the opposite strands. In this study, we updated the TAfinder prediction pipeline to version 2.0, enabling the prediction of all types of TA loci in addition to type II (Supplementary Figure S2).

In brief, the prediction of RNA homologs by BLASTn was added to the pipeline. The protein homolog search module and operon structure detection module remain the same as the original version for types II to VII TA loci prediction. Notably, for the identification of type I TA loci, the toxin gene and antitoxin RNA must be located on opposite DNA strands, rather than forming an operon structure on the same strand. In addition, for the identification of type VIII TA loci, we took into consideration the characteristics of the two experimental validated type VIII TA loci: *creTA*, where the toxin and antitoxin RNAs are located either on the same strand or the opposite strands (19,36,37), and SdsR-RyeA, where the two RNAs are located on opposite strands (38). Accordingly, we made predictions for these two distinct type VIII TA loci based on their respective characteristics. The BLAST reference dataset comprises all the TADB 3.0-archived experimentally validated proteins and RNAs of TA loci archived in TADB 3.0. In addition, in comparison with the previous version of TAfinder, we have expanded the number of HMM profiles for the toxin domains from 108 to 207, and for the antitoxin domains, the number has increased from 201 to 228.

To perform TA loci prediction on completely sequenced bacterial and archaeal genomes using TAfinder 2.0, we first downloaded 34 257 bacterial and 532 archaeal complete genomes from the NCBI RefSeq database (39) on 10 July, 2023, including 36 751 chromosomes and 40 457 plasmids. Subsequently, we used these genome sequences and annotated protein-coding sequences as inputs to TAfinder 2.0 for TA loci prediction (Supplementary Figure S3). To ensure the high specificity of TA loci prediction results, we only kept the proteins and nucleotides with BLAST-based *H*-value > 0.36 (40) as candidate toxins and antitoxins. In addition, the criteria for the length of toxin or antitoxin proteins were set to range between 30 and 500 amino acid residues. Except for the type I TA loci, the intergenic distance between the putative toxin and antitoxin genes on the same DNA strand was set to –20 to 150 bp. For type I TA loci prediction, the maximum distance between the toxin gene and the antisense antitoxin sRNA is set to 200 bp, without any limitation of overlap length. For the prediction of type VIII TA loci, we applied distinct criteria: the maximum distance between toxin RNA and antitoxin RNA was set to 200 bp for *creTA* while this distance was set to <0 bp for SdsR-RyeA.

### Identification of MGEs associated with TA loci

In this study, TA-associated MGEs are defined as the MGEs harboring TA loci or the ISs/transposons flanked by TA loci with an interval < 5 kbp (Supplementary Figure S3). To identify MGEs associated with both experimentally validated and *in silico* predicted TA loci, the MGE identification pipeline VRprofile2 (41) was first utilized to identify MGEs in bacterial genomes, including prophages, GIs, ICEs, integrons, ISs/transposons and IS clusters. The genomic locations of TA loci and predicted MGEs were then analyzed to identify TA-associated MGEs. Pairwise Mash distances (42) of these MGEs were also calculated, and MGEs with Mash distances < 0.01 were considered similar MGEs.

TA-associated MGEs were displayed on the ‘Browse’ page, ‘Statistics’ page and the detailed information page of TA loci. In the ‘Statistics’ page and detailed information pages of TA loci, TA-associated MGEs, the corresponding similar MGEs, and all TA loci harbored by these MGEs are dynamically presented as interactive networks utilizing ECharts (25). Users can access comprehensive details about each MGE by simply clicking on MGE nodes in the network graphs or via MGE buttons available in tables on the ‘Browse’ page. Besides, within the detailed information page for MGEs, cargo genes including TA pairs, antibiotic resistance genes and virulence genes are visually displayed through gene structure plots by SVGene (https://github.com/kblin/svgene). In addition, comparative analysis and interactive visualization of a group of similar MGEs were performed by clinker and clustermap.js (43).

## Results and discussion

Compared to TADB 2.0, the updated TADB 3.0 database offers three major improvements: (i) manual curation of >500 types I to VIII TA loci with experimental evidence and support; (ii) collection of the TAfinder 2.0*-*predicted TA loci in > 34000 completely sequenced prokaryotic genomes; (iii) graphical representation of the relationships of the TA loci and MGEs.

### TADB 3.0 browse module for types I to VIII TA loci

TADB 3.0 provides a flexible and user-friendly web interface with several new features incorporated (Figure 1). Specifically, the ‘Statistics’ module was developed for visualization of the relationships between TA loci and MGEs, the distribution of TA loci across taxonomies and the homology networks of toxin and antitoxin proteins (Supplementary Figure S1). On the ‘Browse’ page and in the detailed information pages dedicated to various types of TA loci, users can access comprehensive details about the strains, TA families, genomic contexts, protein domains and associated MGEs (Figure 1B–F). For instance, the *relBE* family TA locus (*VCA0503/VC_RS15830-VCA0504/VC_RS15835*) is one of the TA loci previously characterized in the chromosome II of *Vibrio cholerae* O1 biovar El Tor str. N16961 (18). This TA locus is located within a superintegron and plays a crucial role in stabilizing this MGE. Meanwhile, as identified by VRprofile2 (41), the TA locus is also flanked by a putative IS*5* family transposase within a 5 kbp distance, supporting the idea that the TA locus might contribute to the stability of its adjacent region (Figure 1F). Visualizing the 5 kbp flanking region of this IS*5* family transposase reveals its connections with three additional TA loci, apart from *VCA0503-VCA0504* (Figure 1G). Moreover, by analyzing other predicted TA loci and MGEs in *V. cholerae*, we found that superintegrons and transposases are widespread in *V. cholerae* genomes, which also encode or are flanked by several putative TA loci, as previously described by Iqbal *et al.* (18) (Figure 1F). Comparison of the flanking genomic regions of these similar transposases indicated that these regions are conserved among a certain amount of *V. cholerae* strains being analyzed (Figure 1H).

### Rigorous prediction of types I to VIII TA loci in prokaryotic genome sequences

In recent years, there has been a substantial increase in both the number of experimentally validated TA pairs and the availability of prokaryotic genome sequencing data. This growing wealth of information necessitates the need to obtain relatively high confidence predicted TA loci dataset to support further experimental research. With the experimentally validated types I to VIII TA loci available in the TADB 3.0 database (Supplementary Table S1), we have upgraded our type II TA loci prediction tool, TAfinder, to version 2.0 (Supplementary Figure S2). This new version is capable of predicting types I to VIII TA loci by utilizing the newly collected experimentally validated data from the TADB 3.0 database. For the prediction of TA loci in completely sequenced prokaryotic genomes, a total of 34 257 bacterial and 532 archaeal completely sequenced genomes were downloaded from the NCBI RefSeq database (39). Subsequently, TAfinder 2.0 was employed to predict TA loci in these genome sequences. To enhance the specificity and reliability of our predictions, we adopted a rigorous methodology that exclusively used experimentally validated TA pairs as the reference dataset and implemented a stringent strategy (Supplementary Figure S3). Briefly, only toxin and antitoxin BLAST hits with *H*-value >0.36 (40) were retained. As a result, our predictions yielded a total of 211 697 TA loci homologs in 34 789 completely sequenced prokaryotic genomes, including 16 009, 168 794, 55, 16 685, 2 786, 2, 388 and 6 978 TA homologs of types I to VIII TA loci, respectively (Supplementary Table S1).

The species with the highest number of *in silico* predicted TA loci for each type and the distribution of TA families within each TA type for each species were displayed in Supplementary Figure S4. As the most extensively studied type of TA systems, type II TA loci homologs are the most abundant among all types of TA loci homologs, partially due to the large number of experimentally validated type II loci used for prediction. Among the species harboring type II TA loci homologues, 99.1% (2769/2795) of the analyzed *Escherichia coli* genomes harbored 39 324 putative type II TA loci in both chromosomes and plasmids, with around 14 type II TA loci per strain (Supplementary Figure S4B). The pronounced representation of type II TA loci among *E. coli* can be attributed, in part, to the extensive availability of completely sequenced *E. coli* genomes. Apart from that, *Mycobacterium tuberculosis* strains possess an average of >50 TA loci, significantly outnumbering other species. Meanwhile, the average number of putative type II TA loci per strain is high across several clinical pathogens, such as *V. cholerae* (17.3) and *Klebsiella pneumoniae* (11.3). Among the predicted type II TA loci, the TA families *vapBC*, *higBA, relBE* and *mazEF* are widespread in the top 20 species with the highest number of predicted type II TA loci, while some other families such as *hipBA* appear to be abundant in certain species.

The predicted TA loci have been cataloged in the TADB 3.0 database available for browsing and download. However, it is noteworthy that the predicted outcomes might not accurately represent the distribution of TA loci and TA families across prokaryotes, but rather serve as high-confidence predictions for reference purposes, highlighting potential TA loci worthy of further experimental validation and investigation. Furthermore, for users specifically interested in particular bacterial strains, it is highly recommended to utilize TAfinder 2.0 with less strict thresholds for TA loci prediction.

### Investigation of the TA-MGE relationships based on genomic location

**Figure 2.**
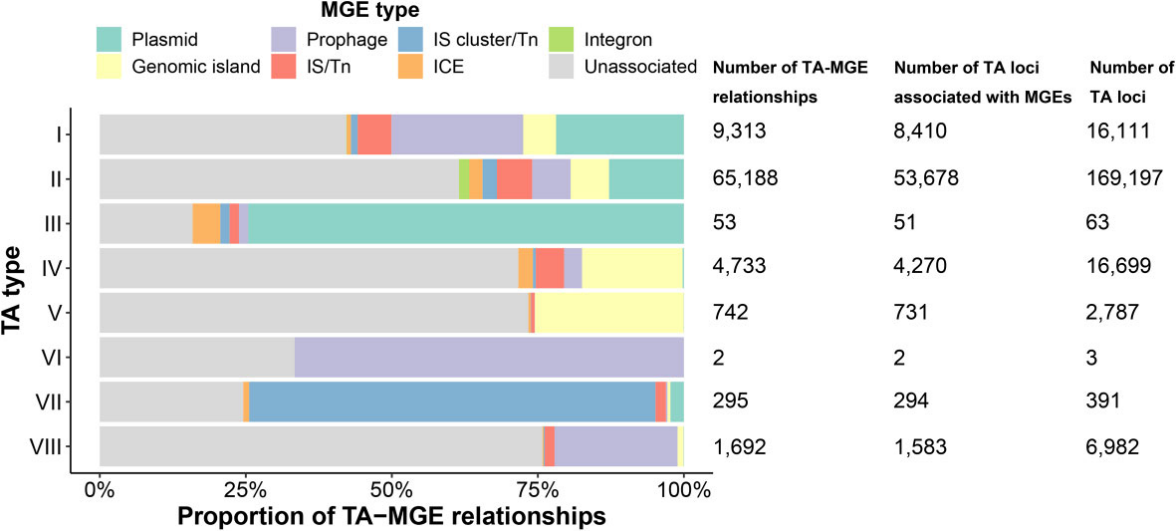
Relationships of the TADB-archived TA loci and MGEs of the prokaryotic organisms. TA-associated MGEs are defined as the MGEs harboring TA loci or the ISs/transposons flanked by TA loci with an interval <5 kb in this study. The stacked bar plots display the proportion of TA-MGE relationships of each TA type. If one TA locus was associated with multiple MGEs or one MGE was associated with multiple TA loci, the relationships were calculated separately. The number of TA-MGE relationships, the number of TA loci associated with MGEs and the total number of TA loci were listed on the right side of the bar plot.

TA pairs are proposed to play a role in maintaining MGEs that contain these genetic modules (13,16–18). After obtaining experimentally validated TA loci together with the *in silico* predicted TA loci, we then utilized VRprofile2 (41) to identify various types of MGEs in TA-harboring prokaryotic strains (Supplementary Figure S3). In this study, the TA-associated MGEs are defined as the MGEs harboring TA loci or the ISs/transposons flanked by TA loci with an interval <5 kb (Supplementary Figure S3). As a result, out of the 211 697 TA loci archived in TADB 3.0, 69 019 (32.6%) TA loci were predicted to be associated with 60 898 MGEs, resulting in a total of 82 018 TA-MGE relationships (Figure 2, Supplementary Table S2 and Supplementary Figure S5). Among various types of MGEs, prophages and plasmids exhibited the highest frequency of relationships with TA loci. Out of 40 457 plasmids being analyzed, 14 163 (35.0%) were found to encode at least one TA locus. Besides, genomic islands and ISs/transposons also showed significant associations with TA loci. For type II TA loci, it was observed that 31.7% (53 678/169 197) of them were associated with MGEs, especially plasmids, genomic islands and prophages (Figure 2). Interestingly, a majority of identified type III TA loci (51/63, 81.0%) were found to be associated with MGEs, particularly plasmids. This observation might be attributed to the relatively low number of experimentally validated type III TA loci currently available, coupled with our stringent prediction methodology, which might have resulted in fewer predictions for type III TA loci. Moreover, we also analyzed the distribution of TA families among different types of MGEs (Supplementary Figure S6). Among the TA families, the type II TA family *relBE* has the most connections with MGEs, followed by the type II TA family *higBA* and the type I TA family *hok-sok*. Meanwhile, the type IV TA family *yeeUV* (also known as *cbtA-cbeA*) is also frequently associated with MGEs. Notably, the type II TA family *relBE* is associated with all types of MGEs, whereas a large number of TA loci of type IV TA family *yeeUV* are located within genomic islands but were seldom found on plasmids. However, it is worth noting that, since our MGE prediction focused solely on strains that harbor TA loci, further investigations are needed to determine the proportion of MGEs in each MGE type associated with TA loci.

## Conclusion

Here, we reported a major upgrade of TADB with a primary focus on TA loci and their relationships with MGEs. TADB 3.0 collected and integrated the systematic information of types I to VIII TA loci. Overall, our rigorous analysis, using a robust methodology that incorporates experimentally validated TA loci and stringent threshold settings, has yielded highly specific prediction results of TA loci in completely sequenced prokaryotic genomes. Furthermore, TADB 3.0 provides insightful visualizations of the relationships of the putative TA loci and MGEs. Altogether, these results available in TADB 3.0 provide researchers a solid foundation for further investigations while recognizing the need for future experimental studies to comprehensively explore the distribution and functional significance of TA pairs in prokaryotes.

## Supplementary Material

gkad962_supplemental_file

## Data Availability

TADB 3.0 is freely available at https://bioinfo-mml.sjtu.edu.cn/TADB3/.
