# Supplementary material for: TADB 3.0: an updated database of bacterial toxin–antitoxin loci and associated mobile genetic elements
Source: Nucleic Acids Res. 2023 Oct 28;52(D1):D784–90. doi: 10.1093/nar/gkad962 (PMC10767807; doi:10.1093/nar/gkad962)
Supplement: gkad962_supplemental_file [file gkad962_supplemental_file.pdf]

TADB 3.0: an updated database of bacterial toxin-antitoxin loci and associated mobile genetic elements

SUPPLEMENTARY DATA

Table S1. Statistical summary of experimentally validated and *in silico* TA loci archived in TADB 3.0 compared to previous database versions.

Table S2. Statistical distribution of predicted TA-MGE relationships for each TA type and MGE type.

Figure S1. Statistics module in TADB 3.0.

Figure S2. Workflow of the types I to VIII TA loci prediction tool TAfinder 2.0.

Figure S3. Prediction of types I to VIII TA loci and identification of TA-associated MGEs.

Figure S4. Taxonomic distribution and TA family distribution of the *in silico* predicted types I to VIII TA loci among the prokaryotic organisms archived in TADB 3.0.

Figure S5. The numbers of various types of MGEs associated with TA loci.

Figure S6. Distribution of the TA-MGE relationships among mobile genetic elements.

Table S1. Statistical summary of experimentally validated and *in silico* TA loci archived in TADB 3.0 compared to previous database versions.

Version		Type I	Type II	Type III	Type IV	Type V	Type VI	Type VII	Type VIII
TADB 3.0 (released in 2023)	Exp.	102	403	8	14	1	1	3	4
	Pred.	16,009	168,794	55	16,685	2,786	2	388	6,978
TADB 2.0 (released in 2018)	Exp.	-	105	-	-	-	-	-	-
	Pred.	-	6,088	-	-	-	-	-	-

Abbreviations: Exp.: TA loci derived from experimental data; Pred.: *in silico* predicted TA.

Table S2. Statistical distribution of predicted TA-MGE relationships for each TA type and MGE type.

MGE type	Type I	Type II	Type III	Type IV	Type V	Type VI	Type VII	Type VIII
Plasmid	3,524	21,672	47	36	1	0	9	4
GI	909	11,144	0	2,877	710	0	2	72
Prophage	3,633	11,113	1	509	1	2	1	1,470
IS/Tn	932	10,231	1	812	17	0	7	117
IS cluster/Tn	185	4,170	1	81	2	0	272	13
ICE	117	3,860	3	418	11	0	4	16
Integron	13	2,998	0	0	0	0	0	0

Abbreviations: GI: genomic island; IS: insertion sequence; Tn: transposon; ICE: integrative and conjugative element.

Note: The TA-MGE relationships were calculated separately if one TA locus was associated with multiple MGEs or one MGE was associated with multiple TA loci.

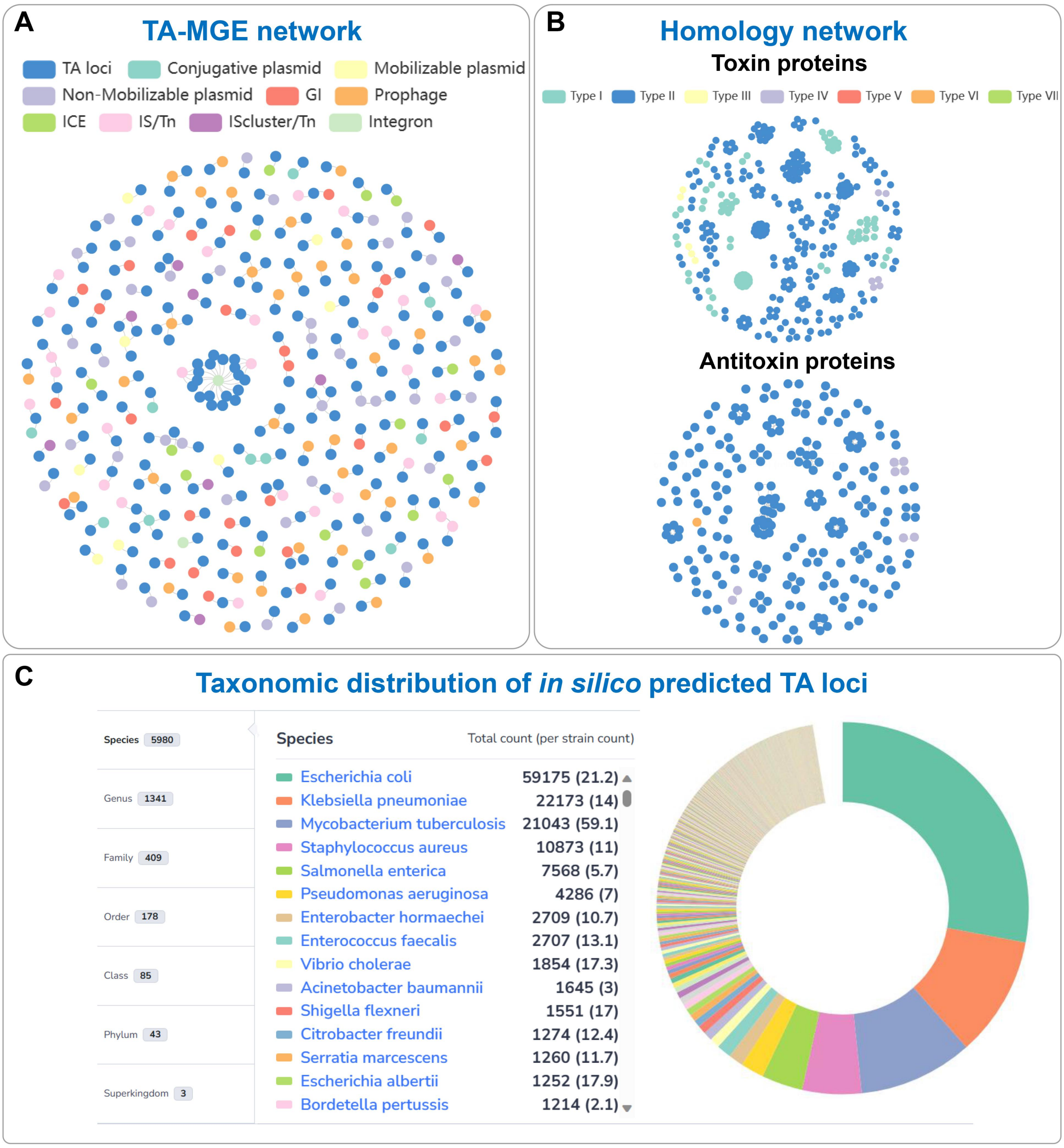

Figure S1. Statistics module in TADB 3.0. **(A)** Interactive networks displaying the TA-MGE relationships. Links between TA loci and MGEs indicate that the TA loci are located within MGEs or the TA loci are flanked by ISs/transposons with an interval <5 kb. Note that only experimentally validated TA loci are displayed. **(B)** Interactive networks displaying the homology networks of different types of toxin proteins and antitoxin proteins. Links between proteins indicate that the BLASTp-based H_a-value between these proteins is greater than 0.36 (1,2). **(C)** Interactive pie charts showing the taxonomic distribution of *in silico* predicted TA loci. Users can browse for TA loci in a specific taxon by clicking on the corresponding pie chart region.

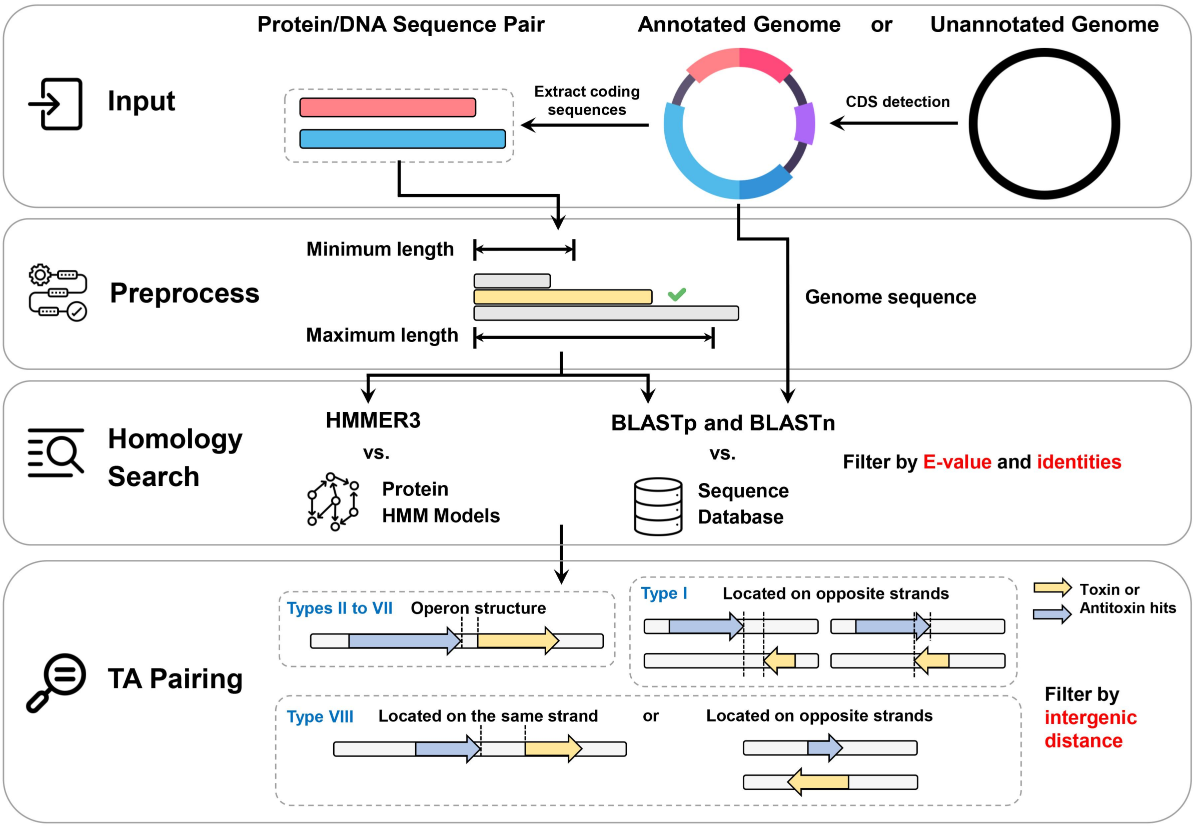

Figure S2. Workflow of the types I to VIII TA loci prediction tool TAfinder 2.0. The updated TAfinder 2.0 contains four main steps for TA loci prediction. For the input section, two types of inputs are acceptable: an annotated genome in the GenBank format or an unannotated genome sequence in the FASTA format. For an unannotated genome sequence input, Prodigal would be used for protein-coding sequence (CDS) identification before sequence extraction (3). In the preprocess section, the extracted sequences would be filtered by user-defined maximum length (500 a.a. by default) and minimum length (30 a.a. by default). In the homology search section, the protein sequences are input to BLASTp and HMMER3 to search for protein homologues, while the genome sequence is input to BLASTn to identify RNA toxins and antitoxins. The E-value (0.01 by default) for BLAST and HMMER3 as well as the identities (30% by default) for BLAST are set to filter out the results. In the TA pairing section, for the identification of types II to VII TA loci, the toxin hits and antitoxin hits should be located in the same strand, and the maximum intergenic distance (150 bp by default) is set for identifying the TA operon structure. For the identification of type I TA loci, the toxin gene and antitoxin RNA should be located on the opposite DNA strands, rather than forming an operon structure on the same strand. In addition, for the identification of type VIII TA loci, we took into consideration the two experimentally validated type VIII systems. The *creTA* loci had RNAs located either on the same strand or on the opposite strands (4-6), while the SdsR-RyeA loci had the two RNAs located on the opposite strands (7). Consequently, we predicted these two distinct type VIII TA loci based on their respective characteristics.

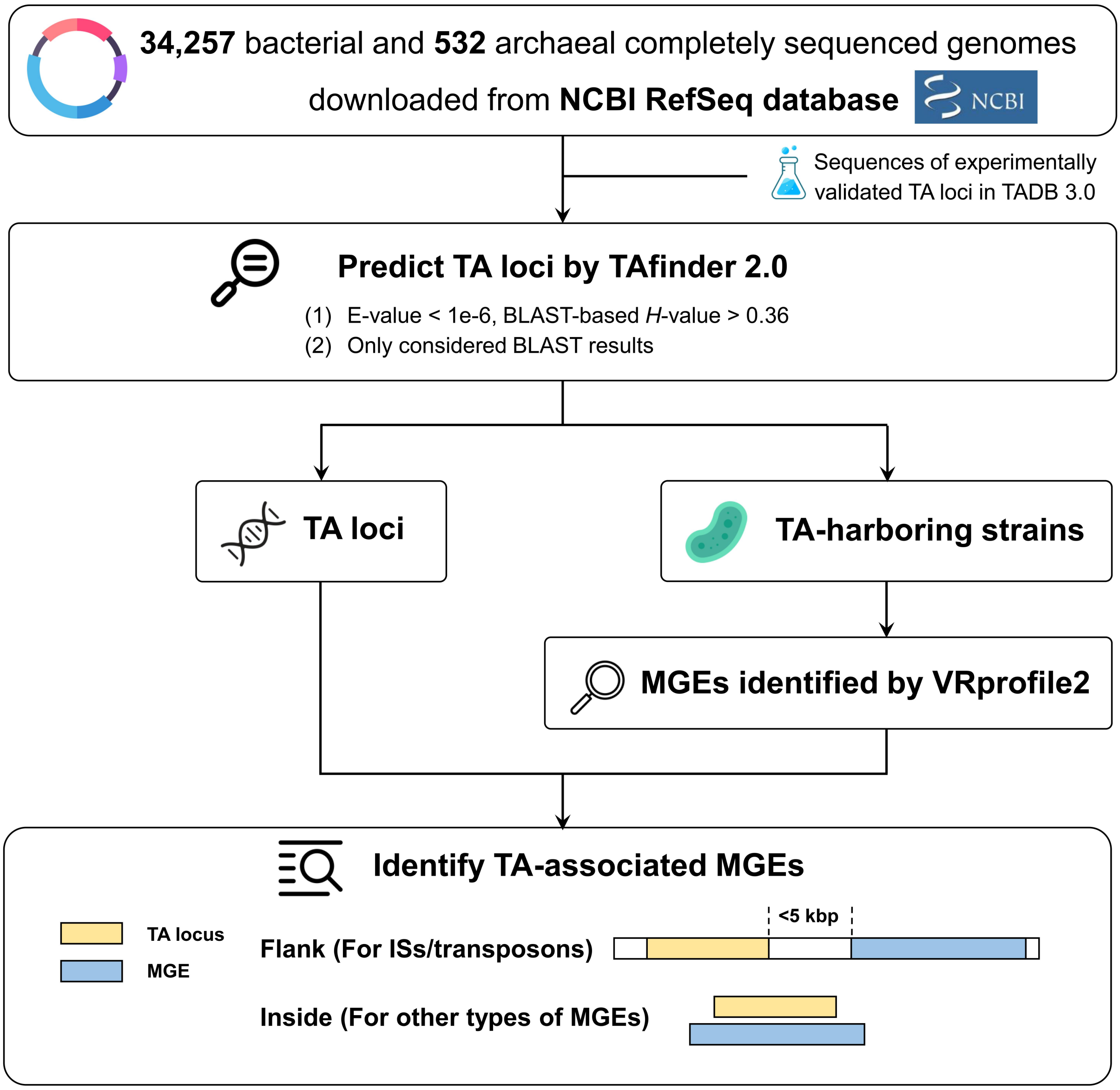

Figure S3. Prediction of types I to VIII TA loci and identification of TA-associated MGEs. TA loci and MGE were predicted using TAfinder 2.0 and VRprofile2, respectively. To increase the specificity and reliability of TA loci predictions, only toxin and antitoxin BLAST hits with *H*-value > 0.36 (2) were kept. TA-associated MGEs are defined as the MGEs harboring TA loci or the ISs/transposons flanked by TA loci with an interval <5 kbp.

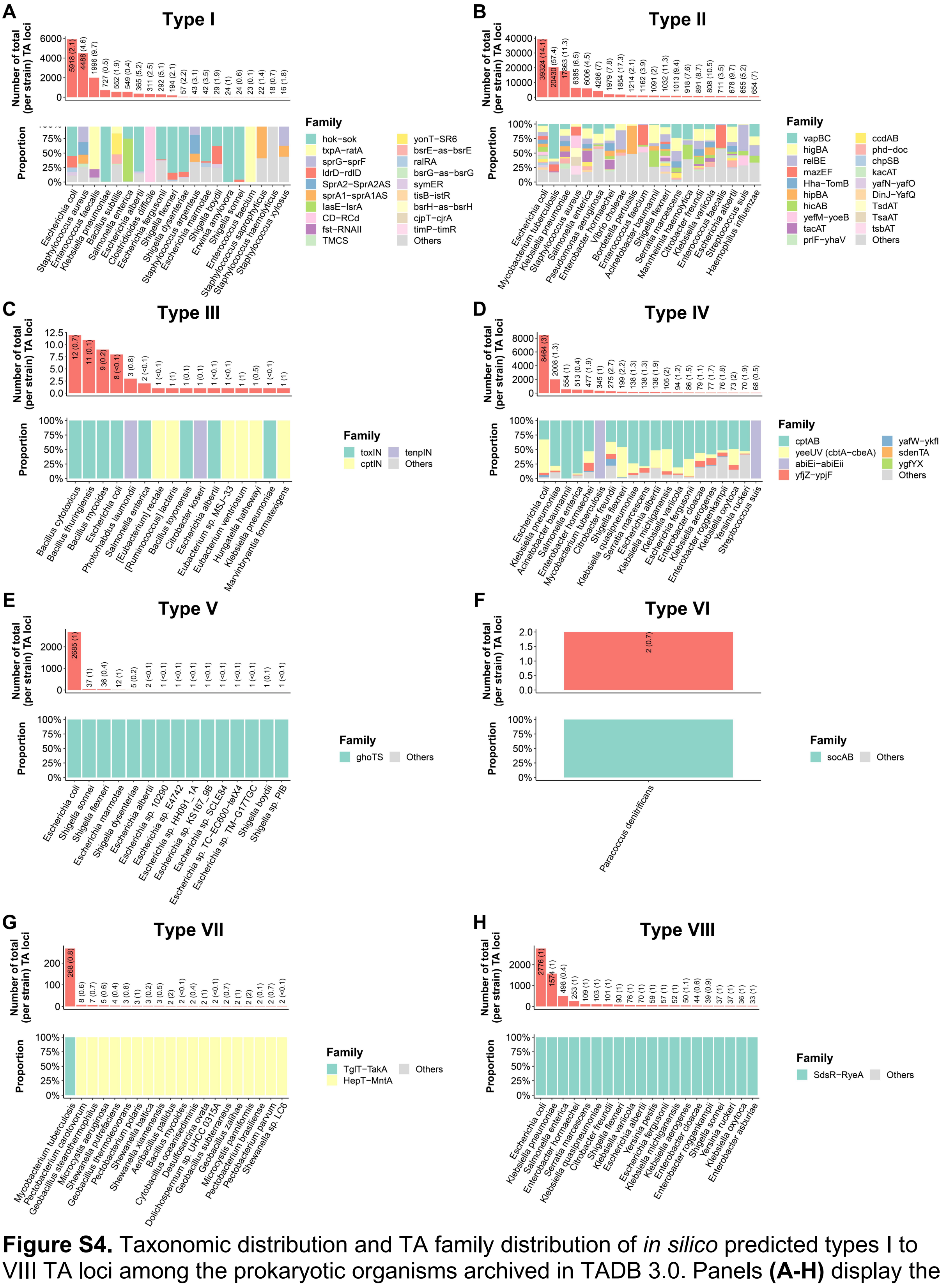

Figure S4. Taxonomic distribution and TA family distribution of *in silico* predicted types I to VIII TA loci among the prokaryotic organisms archived in TADB 3.0. Panels **(A-H)** display the distributions of types I to VIII loci, respectively. The upper bar plots display the number of strains of each species harboring each type of predicted TA loci. The number of TA loci of each type in each species is also displayed in total and per strain. The lower stacked bar plots display the TA family distribution of each type of TA system within each species. Only the top 20 species with the highest numbers of predicted TA loci were displayed for each type of TA system.

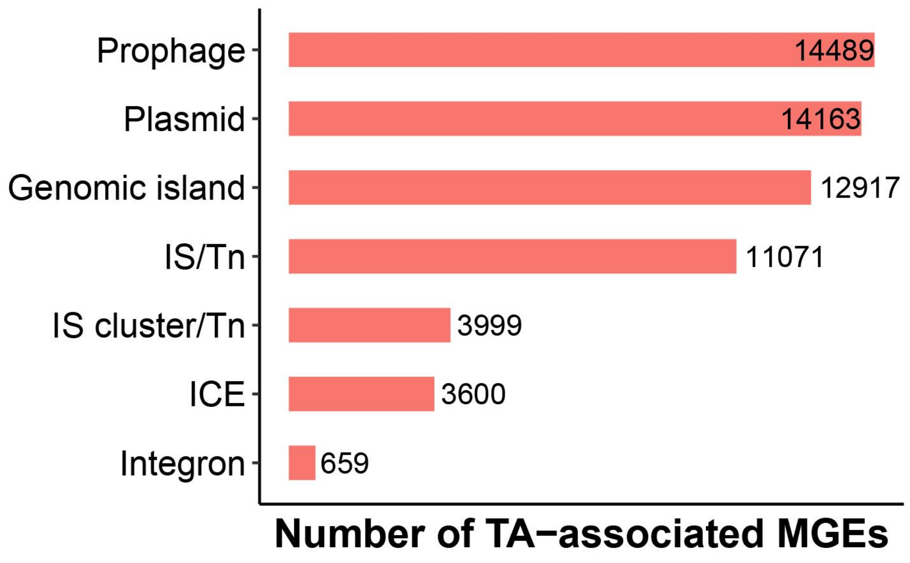

Figure S5. The numbers of various types of MGEs associated with TA loci.

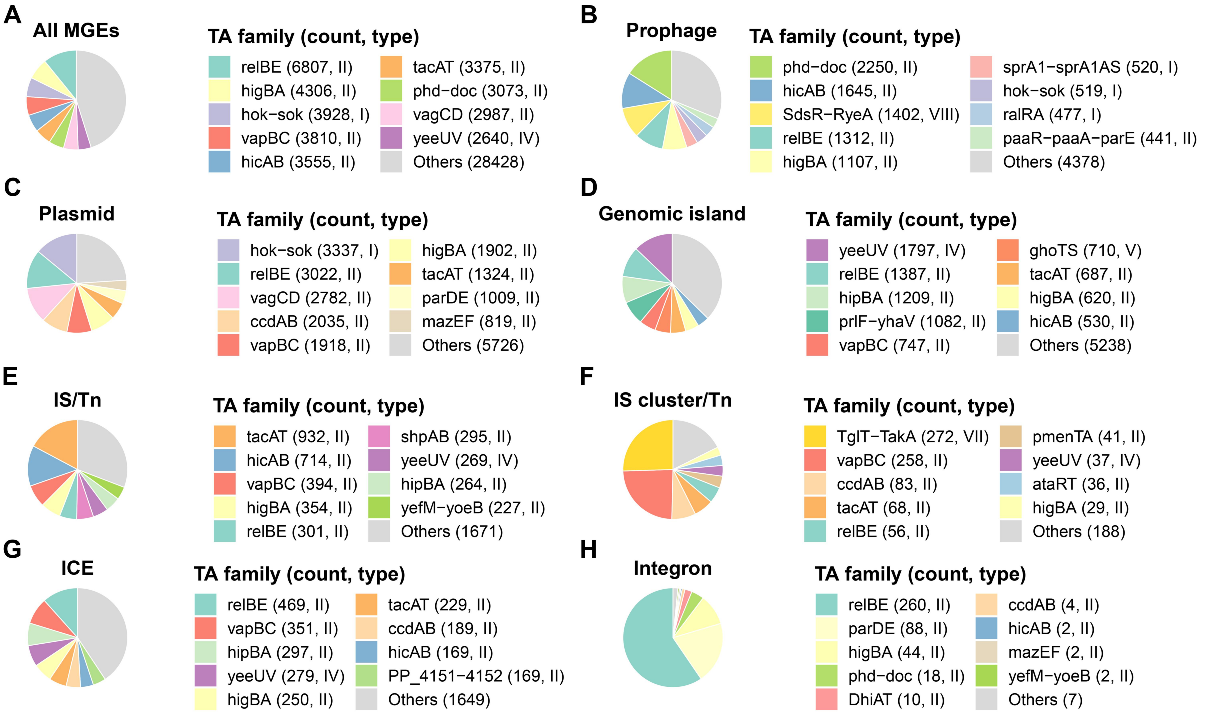

Figure S6. Distribution of the TA-MGE relationships among mobile genetic elements. Panels **(A-H)** show the distributions of TA families in various MGEs, including plasmids, genomic islands, prophages, ISs/transposons (Tn), IS cluster/Tn, integrative conjugative elements (ICEs) and integrons (Supplementary Figure S4), respectively. For each MGE type, only the top 10 associated TA families are displayed. For each TA family, the number of TA-MGE relationships is displayed.

REFERENCES

1. Camacho, C., Coulouris, G., Avagyan, V., Ma, N., Papadopoulos, J., Bealer, K. and Madden, T.L. (2009) BLAST+: architecture and applications. *BMC Bioinformatics*, **10**, 421.
2. Li, J., Tai, C., Deng, Z., Zhong, W., He, Y. and Ou, H.Y. (2018) VRprofile: gene-cluster-detection-based profiling of virulence and antibiotic resistance traits encoded within genome sequences of pathogenic bacteria. *Brief Bioinform*, **19**, 566-574.
3. Hyatt, D., Chen, G.L., Locascio, P.F., Land, M.L., Larimer, F.W. and Hauser, L.J. (2010) Prodigal: prokaryotic gene recognition and translation initiation site identification. *BMC Bioinformatics*, **11**, 119.
4. Li, M., Gong, L., Cheng, F., Yu, H., Zhao, D., Wang, R., Wang, T., Zhang, S., Zhou, J., Shmakov, S.A. *et al.* (2021) Toxin-antitoxin RNA pairs safeguard CRISPR-Cas systems. *Science*, **372**.
5. Cheng, F., Wang, R., Yu, H., Liu, C., Yang, J., Xiang, H. and Li, M. (2021) Divergent degeneration of creA antitoxin genes from minimal CRISPRs and the convergent strategy of tRNA-sequestering CreT toxins. *Nucleic Acids Res*, **49**, 10677-10688.
6. Wang, R., Shu, X., Zhao, H., Xue, Q., Liu, C., Wu, A., Cheng, F., Wang, L., Zhang, Y., Feng, J. *et al.* (2023) Associate toxin-antitoxin with CRISPR-Cas to kill multidrug-resistant pathogens. *Nat Commun*, **14**, 2078.
7. Choi, J.S., Kim, W., Suk, S., Park, H., Bak, G., Yoon, J. and Lee, Y. (2018) The small RNA, SdsR, acts as a novel type of toxin in Escherichia coli. *RNA Biol*, **15**, 1319-1335.
